# Supplementary material for: An ecological examination of early adolescent e-cigarette use: A machine learning approach to understanding a health epidemic
Source: PLoS One. 2024 Feb 14;19(2):e0287878. doi: 10.1371/journal.pone.0287878 (PMC10866513; doi:10.1371/journal.pone.0287878)

Fig S1. Receiver Operating Characteristics (ROC) Curve Estimating Area Under the Curve (AUC) for Models Classifying Exclusive Lifetime E-Cigarette Use. EN = Elastic Net, KNN = K-Nearest Neighbors, NN = Neural Networks, RF = Random Forest, LR = Logistic Regression.

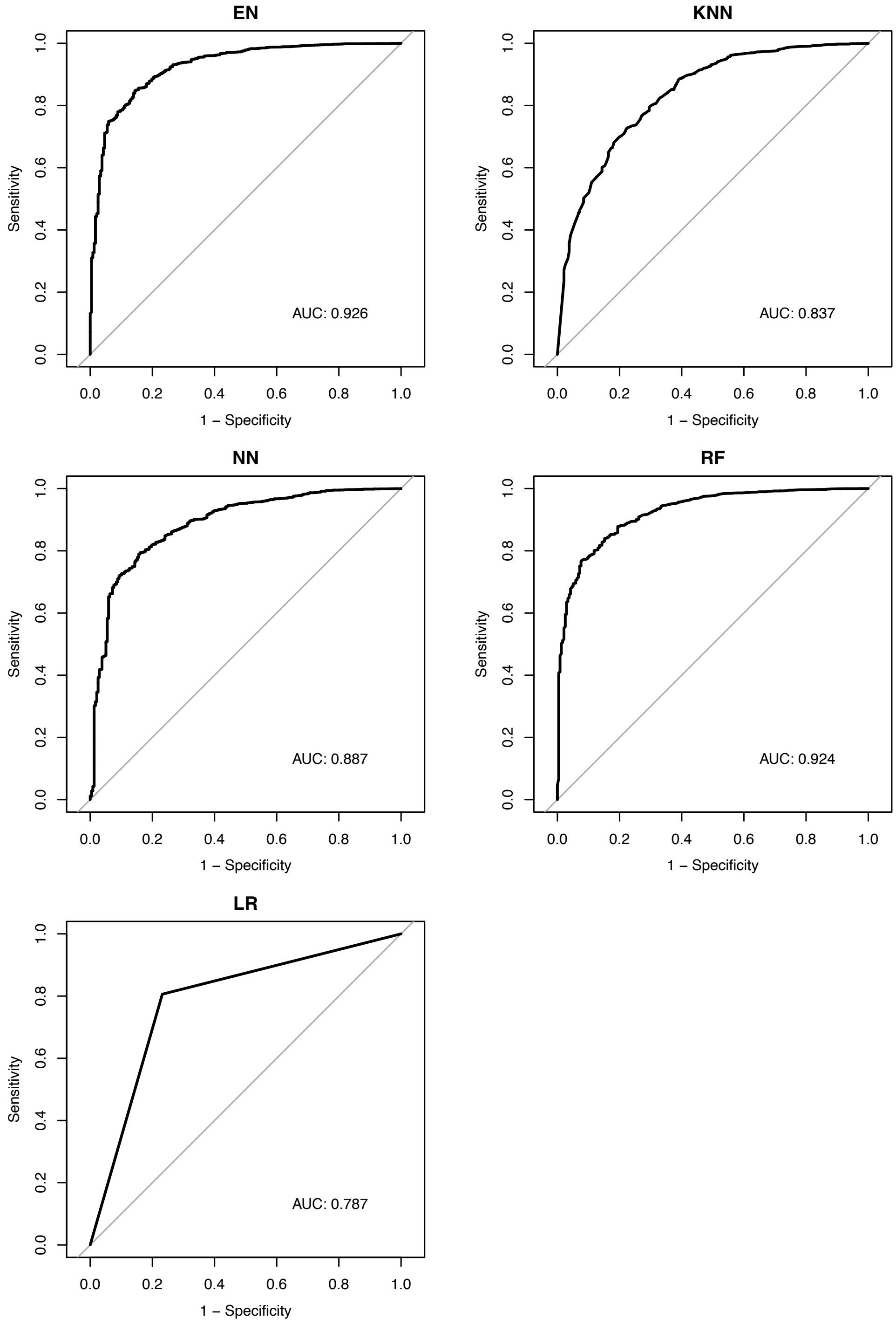

Fig S2. Receiver Operating Characteristics (ROC) Curve Estimating Area Under the Curve (AUC) for Models Classifying Dual Lifetime Tobacco and E-Cigarette Use. EN = Elastic Net, KNN = K-Nearest Neighbors, NN = Neural Networks, RF = Random Forest, LR = Logistic Regression.

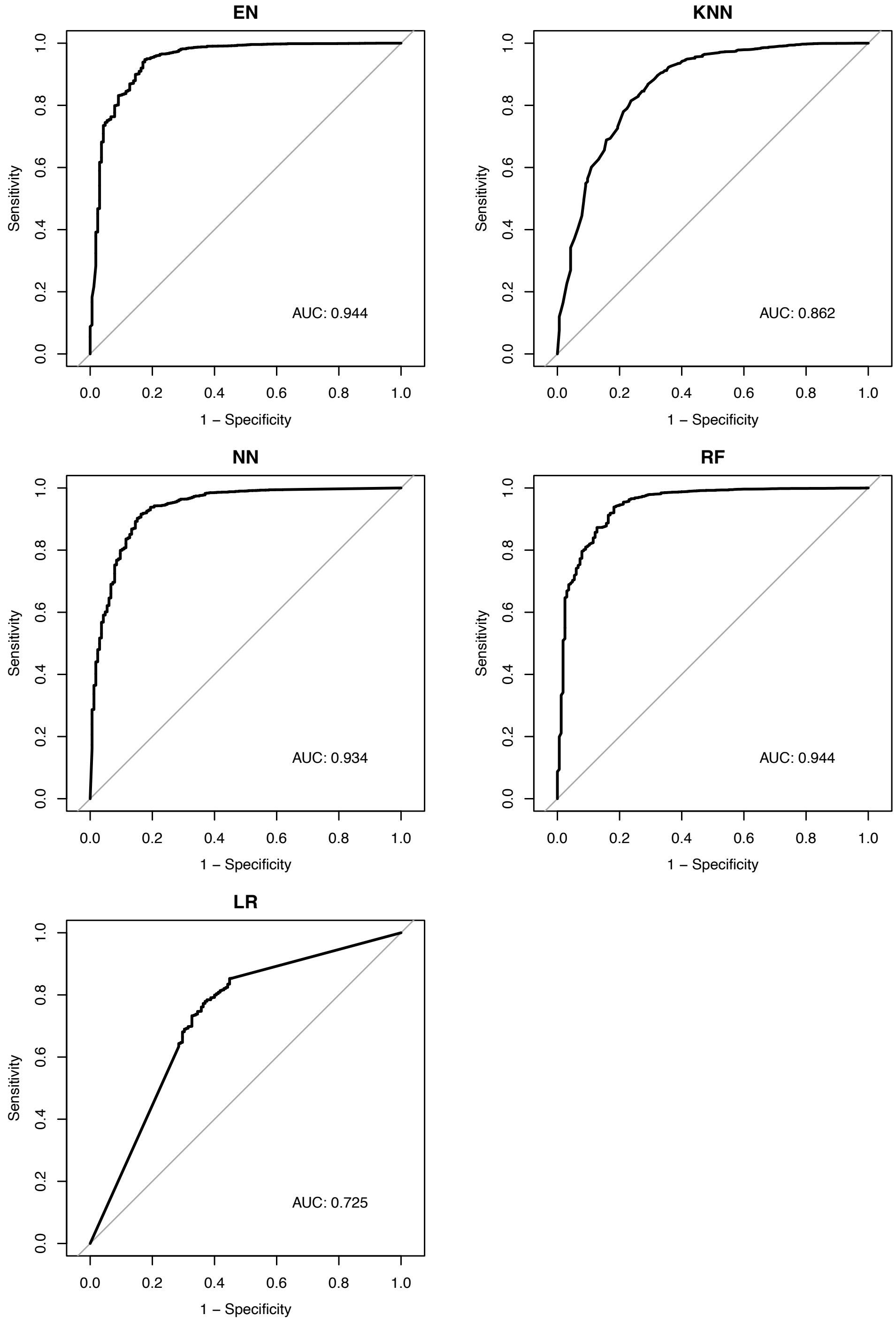

Fig S3. Cross-Tabulation Visualization Showing Proportion of Exclusive Lifetime E-cigarette Use by Perceived Availability of E-Cigarettes. VH = Very Hard, SH = Sort of Hard, SE = Sort of Easy, VE = Very Easy.

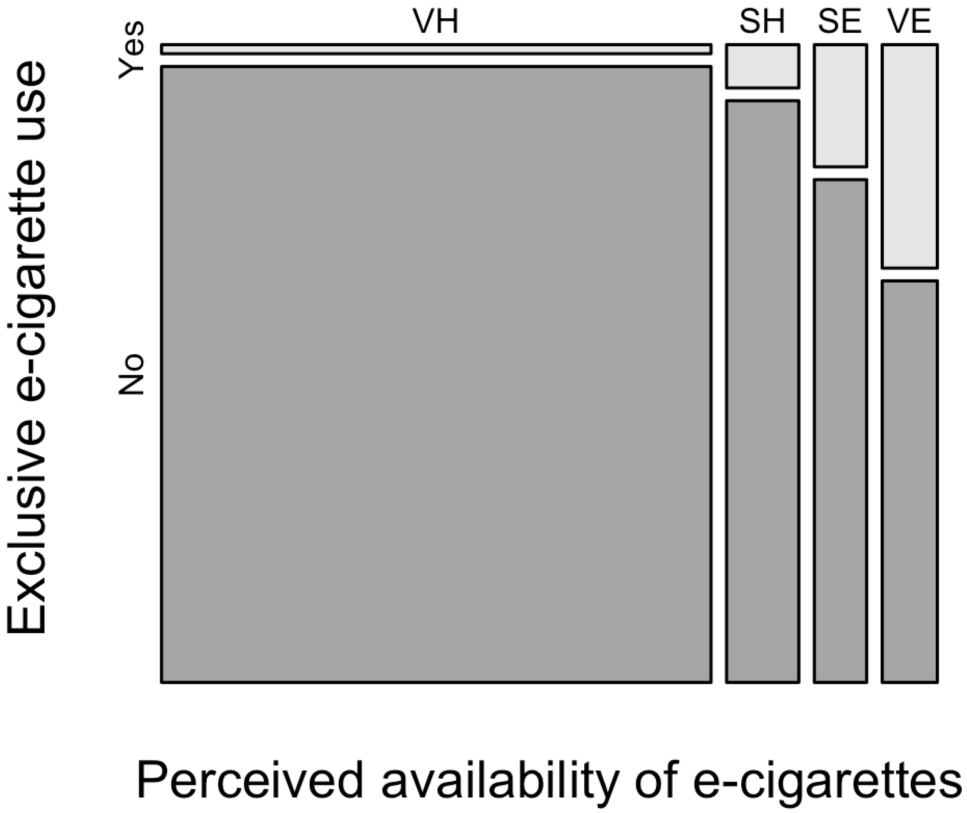

Fig S4. Cross-Tabulation Visualization Showing Proportion of Exclusive Lifetime E-Cigarette Use by Lifetime Alcohol Use.

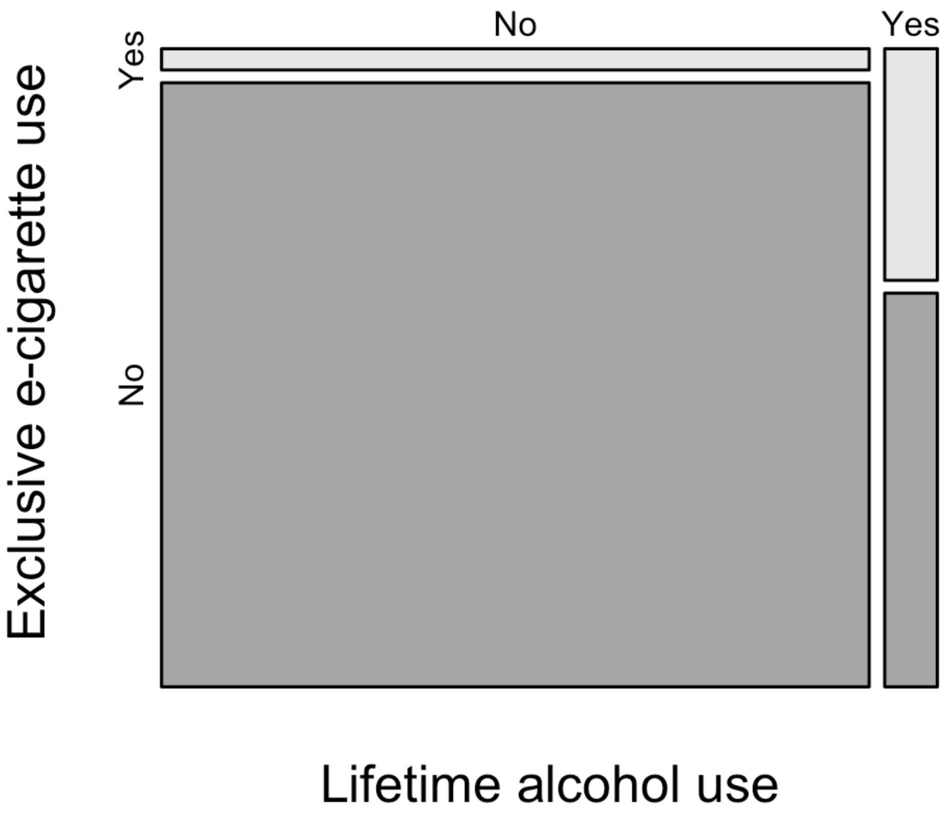

Fig S5. Cross-Tabulation Visualization Showing Proportion of Exclusive Lifetime E-Cigarette Use by Parents Attitudes Regarding their Use of Vape Products. V = Very Wrong, W = Wrong, A = A Little Bit Wrong, N = Not Wrong at All.

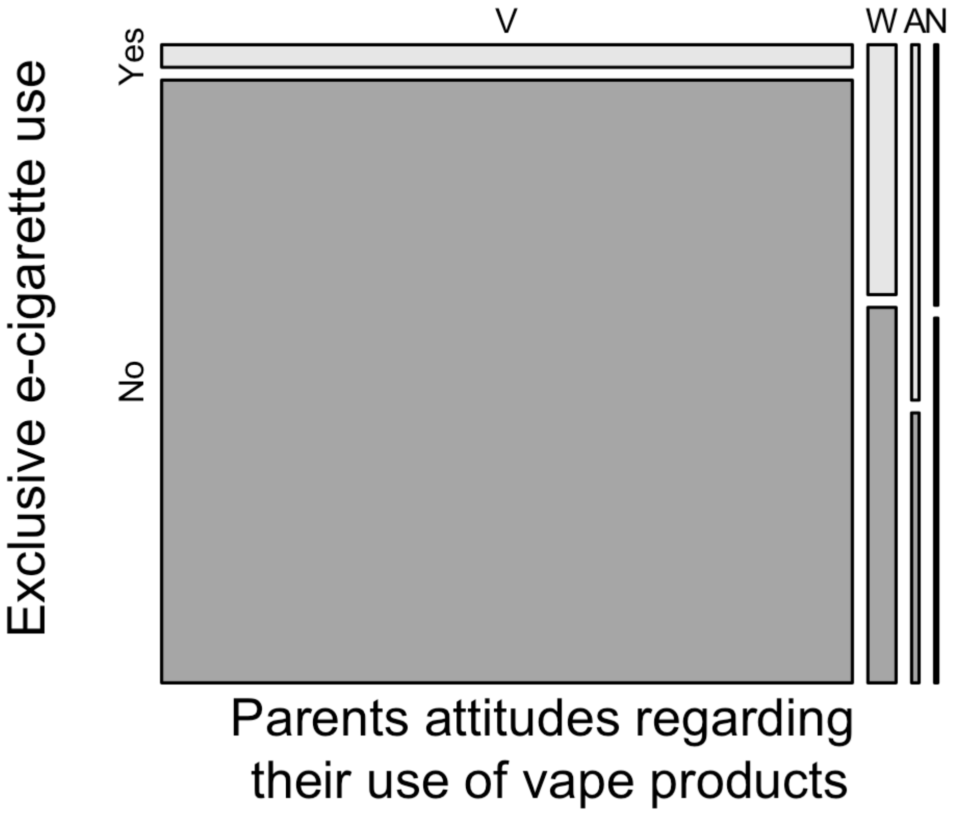

Fig S6. Cross-Tabulation Visualization Showing Proportion of Exclusive Lifetime E-Cigarette Use by School Suspension. 1-2 = 1 or 2 Times, + = 3 or More Times.

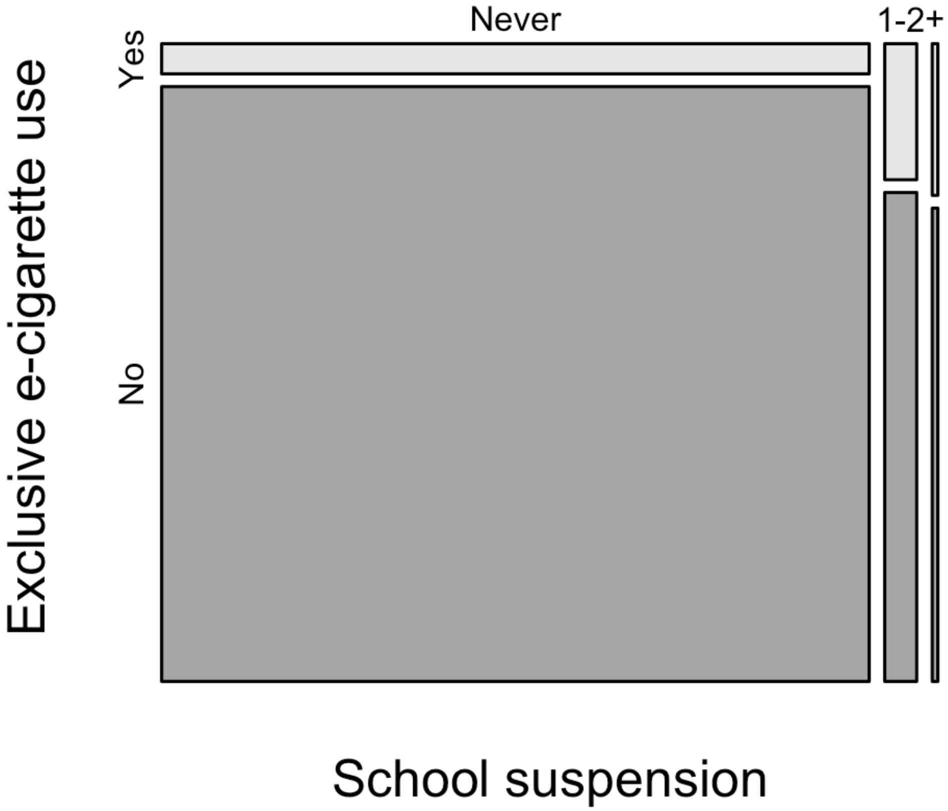

Fig S7. Cross-Tabulation Visualization Showing Proportion of Exclusive Lifetime E-Cigarette Use by Perceived Risk of E-Cigarettes. G = Great Risk, M = Moderate Risk, S = Slight Risk, N = No Risk.

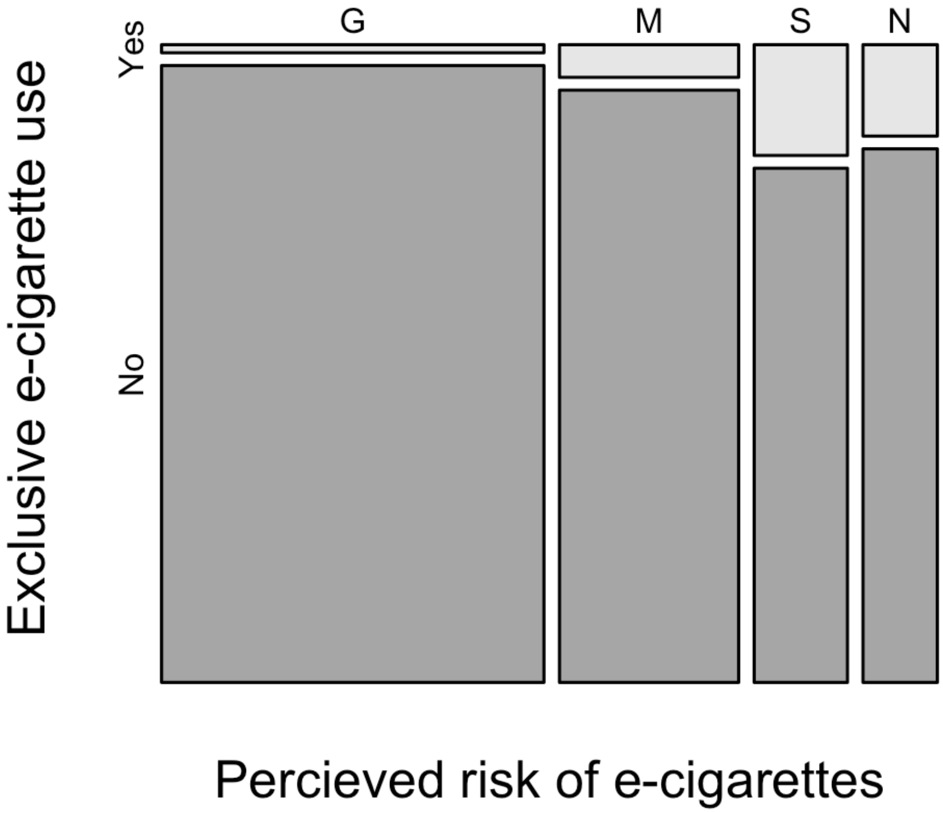

Fig S8. Cross-Tabulation Visualization Showing Proportion of Exclusive Lifetime E-Cigarette Use by Lifetime Marijuana Use.

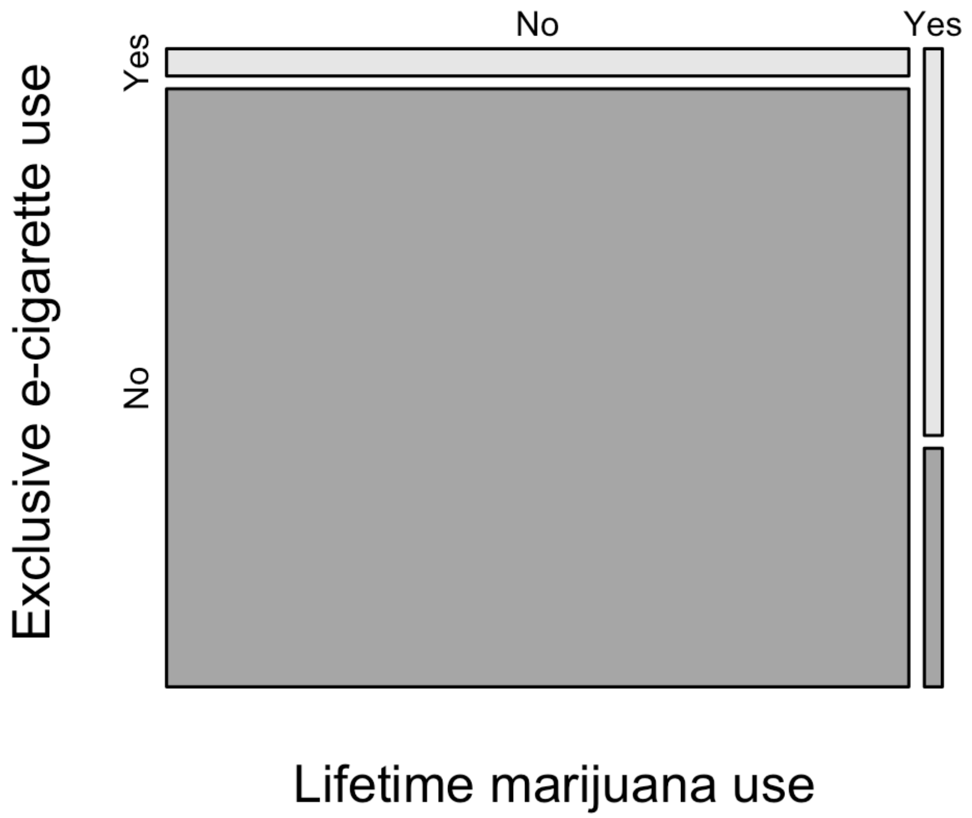

Fig S9. Cross-Tabulation Visualization Showing Proportion of Exclusive Lifetime E-Cigarette Use by Best Friend(s) Tried Alcohol. 1-2 = 1 or 2 Friends, 3-4 = 3 or 4 Friends.

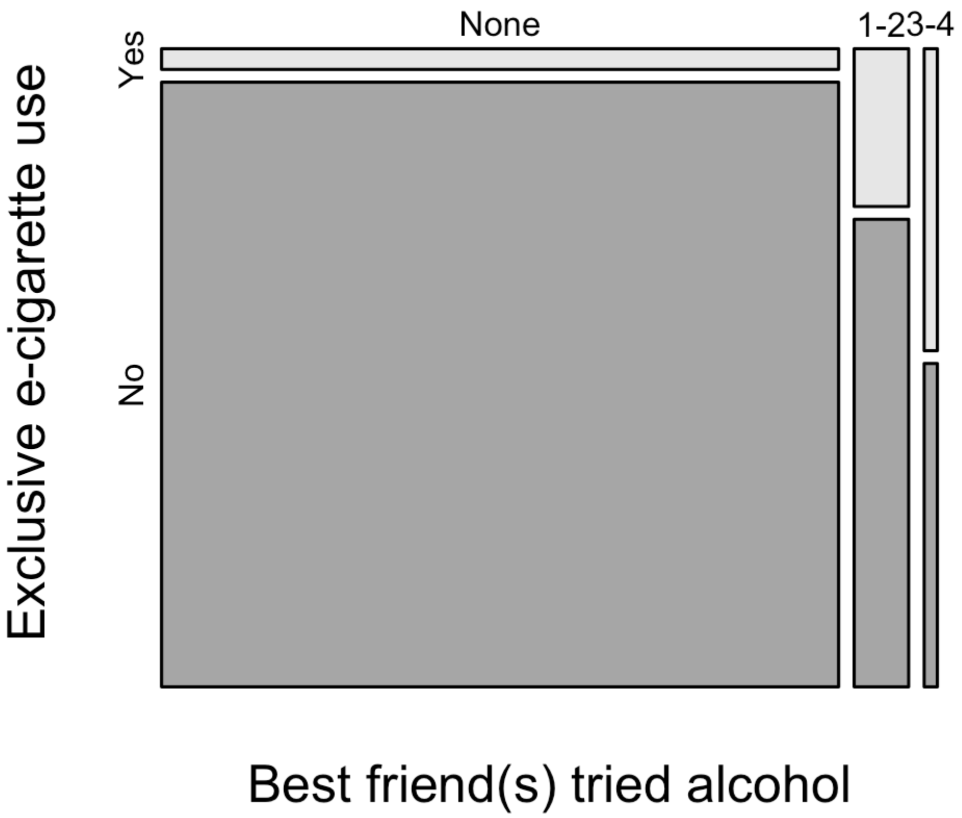

Fig S10. Cross-Tabulation Visualization Showing Proportion of Exclusive Lifetime E-Cigarette Use by Best Friend(s) Used Marijuana. 1-2 = 1 or 2 Friends, 3-4 = 3 or 4 Friends.

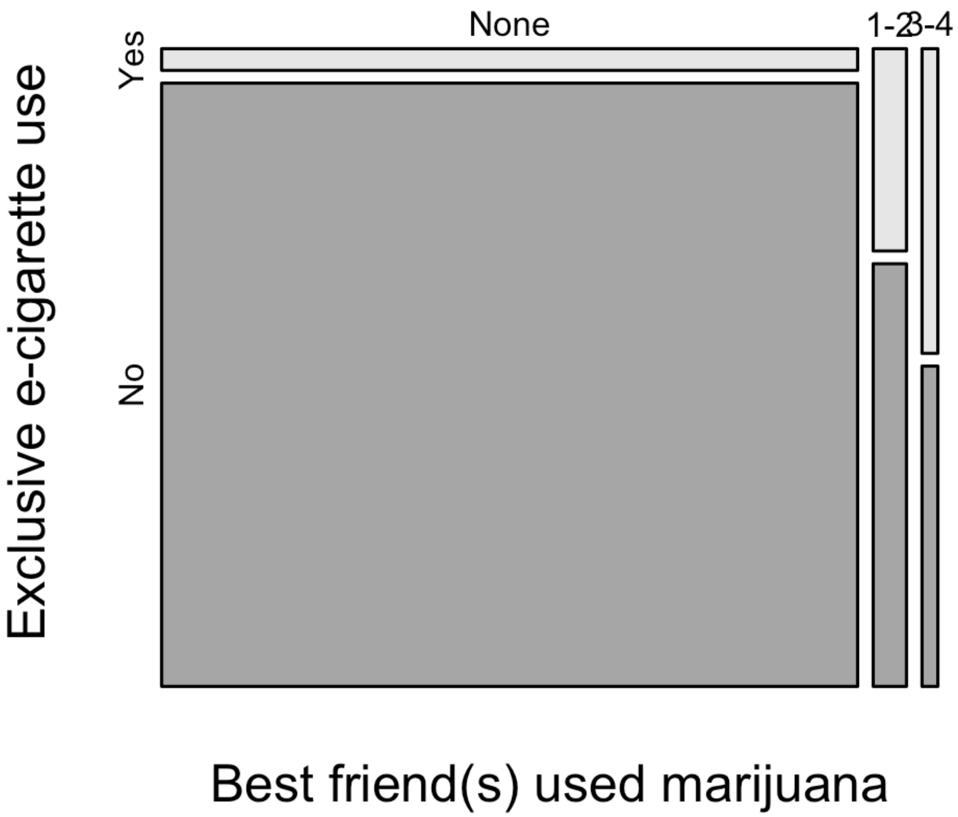

Fig S11. Cross-Tabulation Visualization Showing Proportion of Exclusive Lifetime E-Cigarette Use by Perceived Risk of Smoking Marijuana Regularly. G = Great Risk, M = Moderate Risk, S = Slight Risk, N = No Risk.

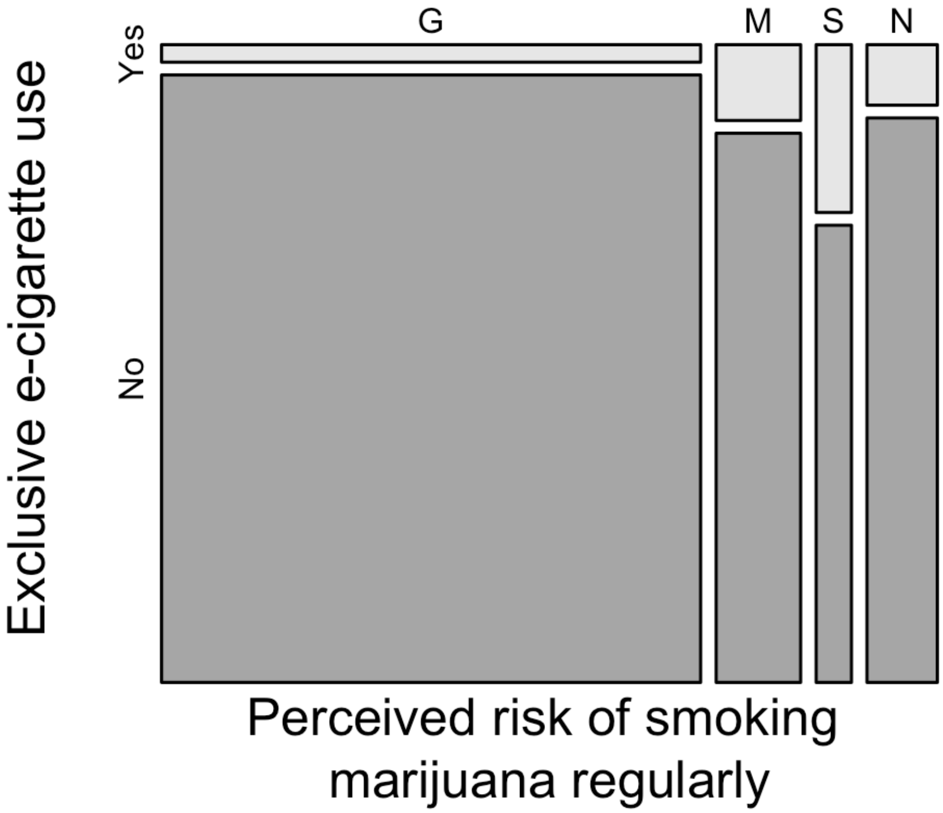

Fig S12. Cross-Tabulation Visualization Showing Proportion of Dual Lifetime Tobacco and E-Cigarette Use by Lifetime Alcohol Use.

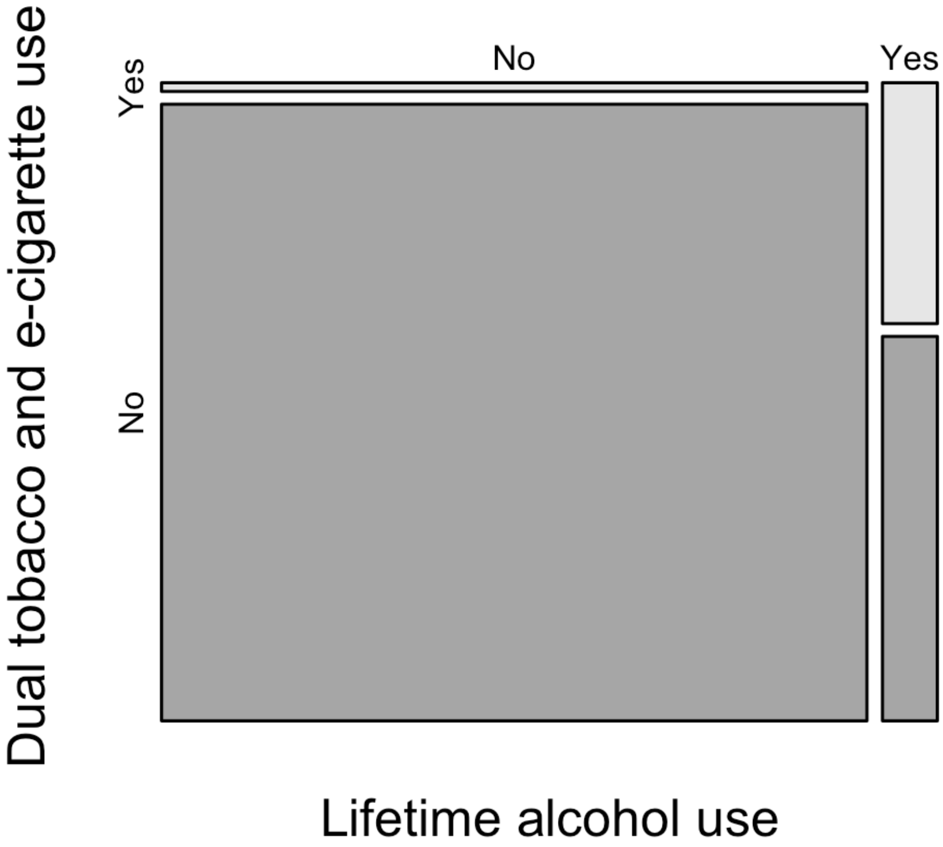

Fig S13. Cross-Tabulation Visualization Showing Proportion of Dual Lifetime Tobacco and E-Cigarette Use by Lifetime Marijuana Use.

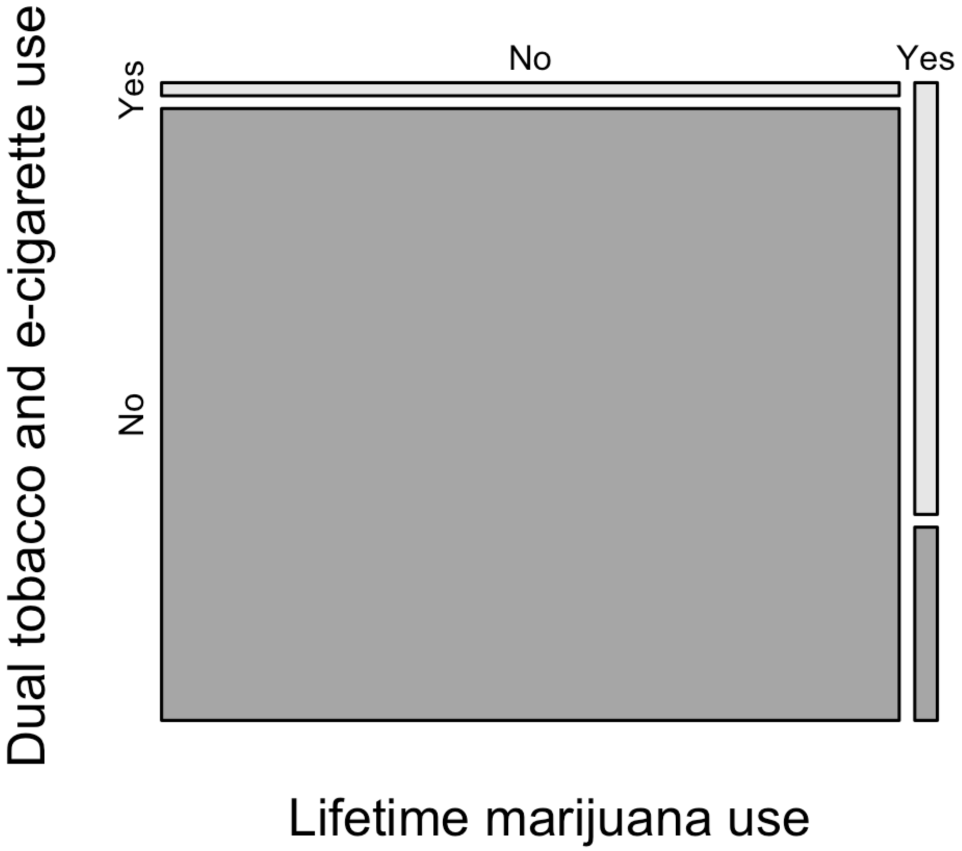

Fig S14. Cross-Tabulation Visualization Showing Proportion of Dual Lifetime Tobacco and E-Cigarette Use by Perceived Availability of E-Cigarettes. VH = Very Hard, SH = Sort of Hard, SE = Sort of Easy, VE = Very Easy.

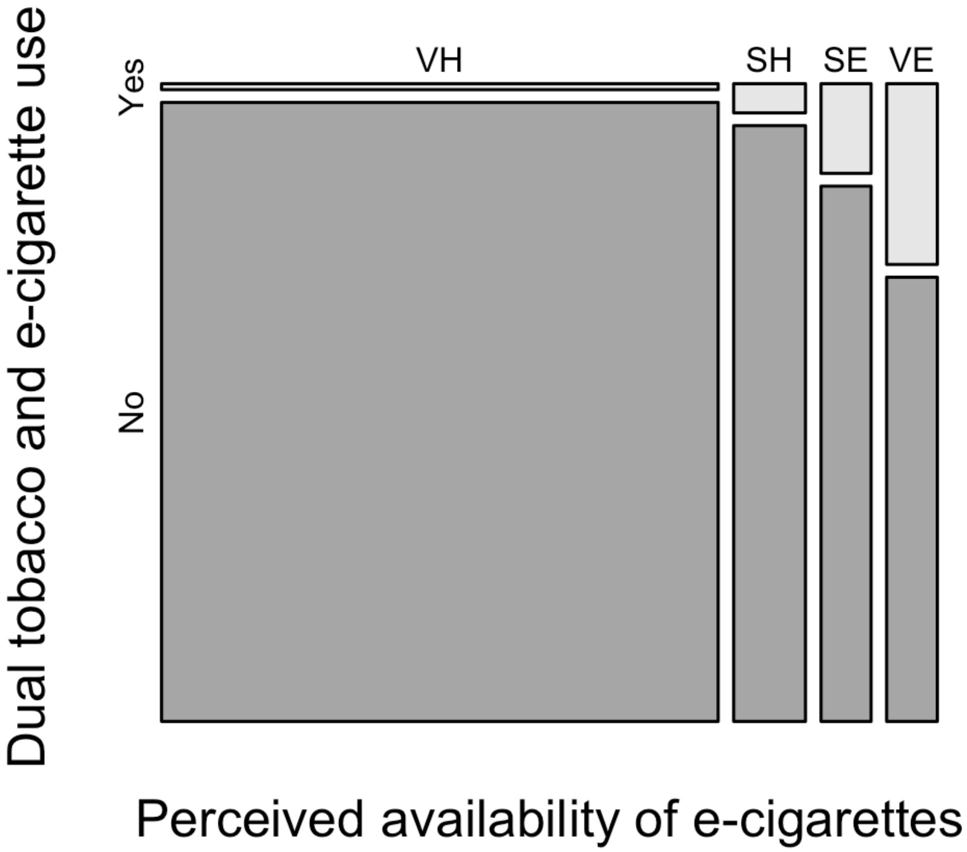

Fig S15. Cross-Tabulation Visualization Showing Proportion of Dual Lifetime Tobacco and E-Cigarette Use by Best Friend(s) Smoked Cigarettes. 1-2 = 1 or 2 Friends, 3-4 = 3 or 4 Friends.

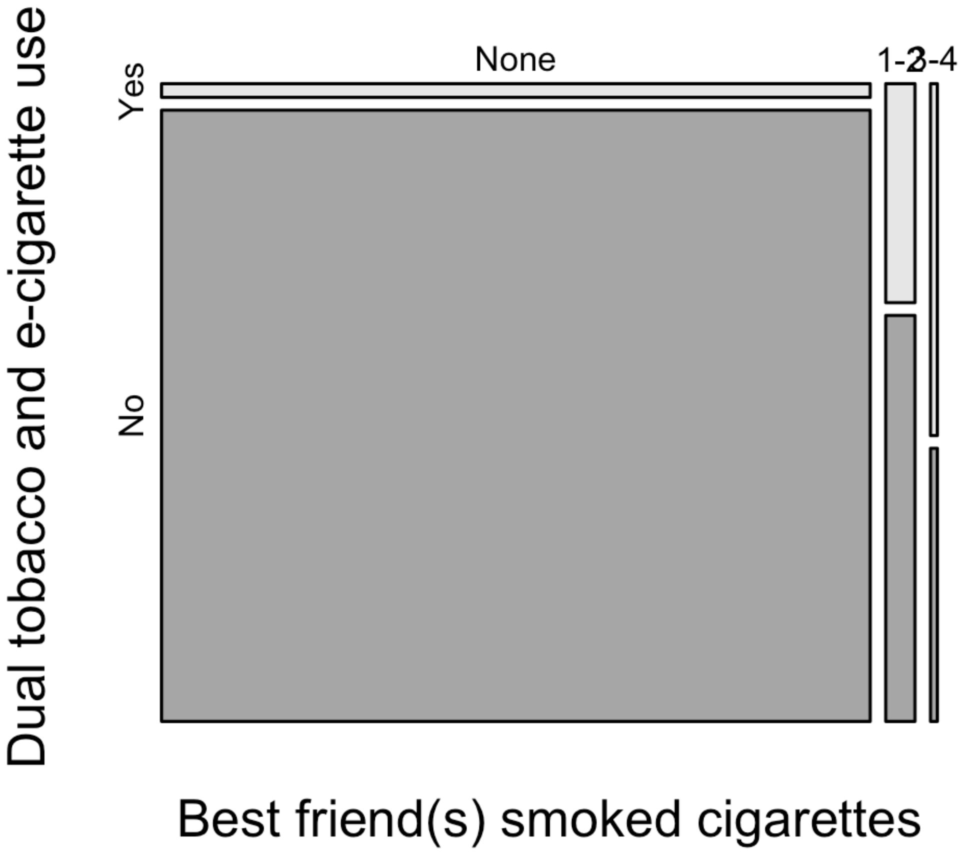

Fig S16. Cross-Tabulation Visualization Showing Proportion of Dual Lifetime Tobacco and E-Cigarette by Perceived Risk of E-cigarettes. G = Great Risk, M = Moderate Risk, S = Slight Risk, N = No Risk.

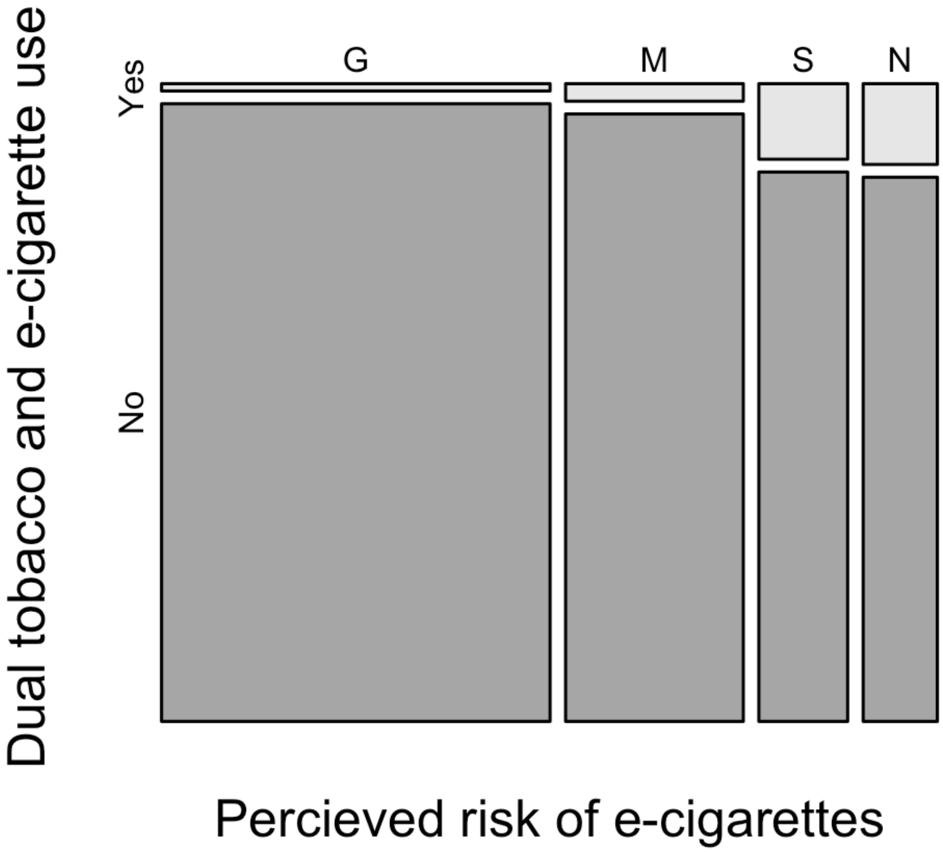

Fig S17. Cross-Tabulation Visualization Showing Proportion of Dual Lifetime Tobacco and E-Cigarette by Lifetime Inhalants Use.

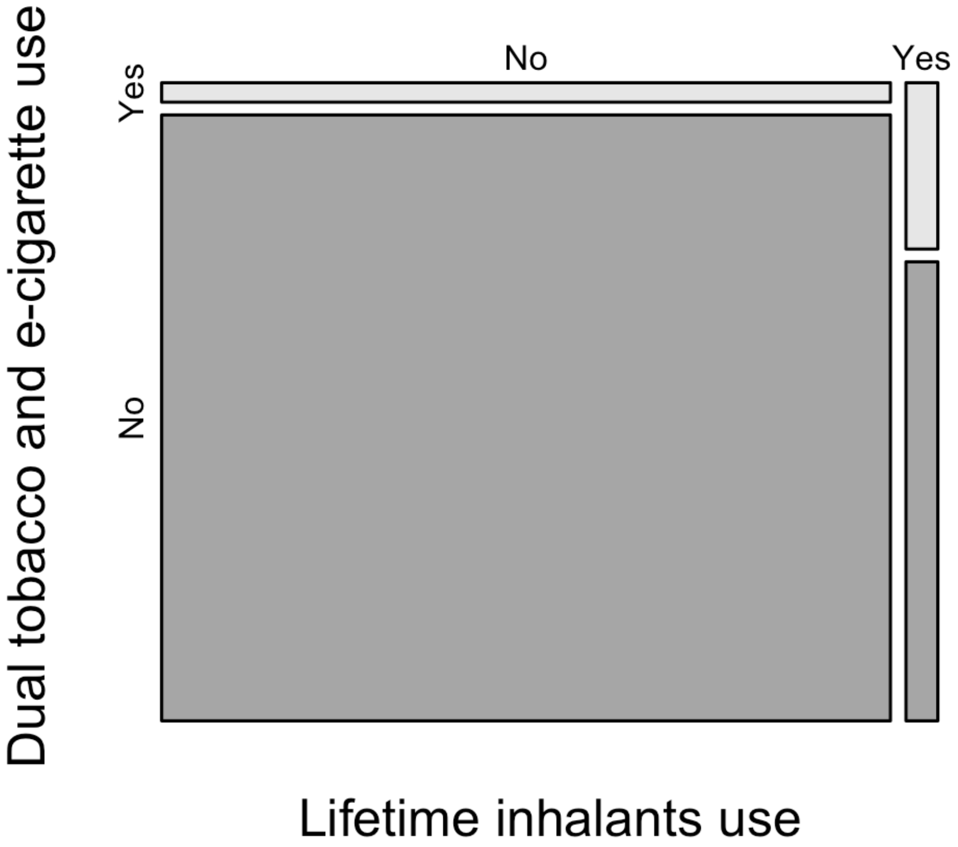

Fig S18. Cross-Tabulation Visualization Showing Proportion of Dual Lifetime Tobacco and E-Cigarette by School Suspension. 1-2 = 1 or 2 Times, + 3 or More Times.

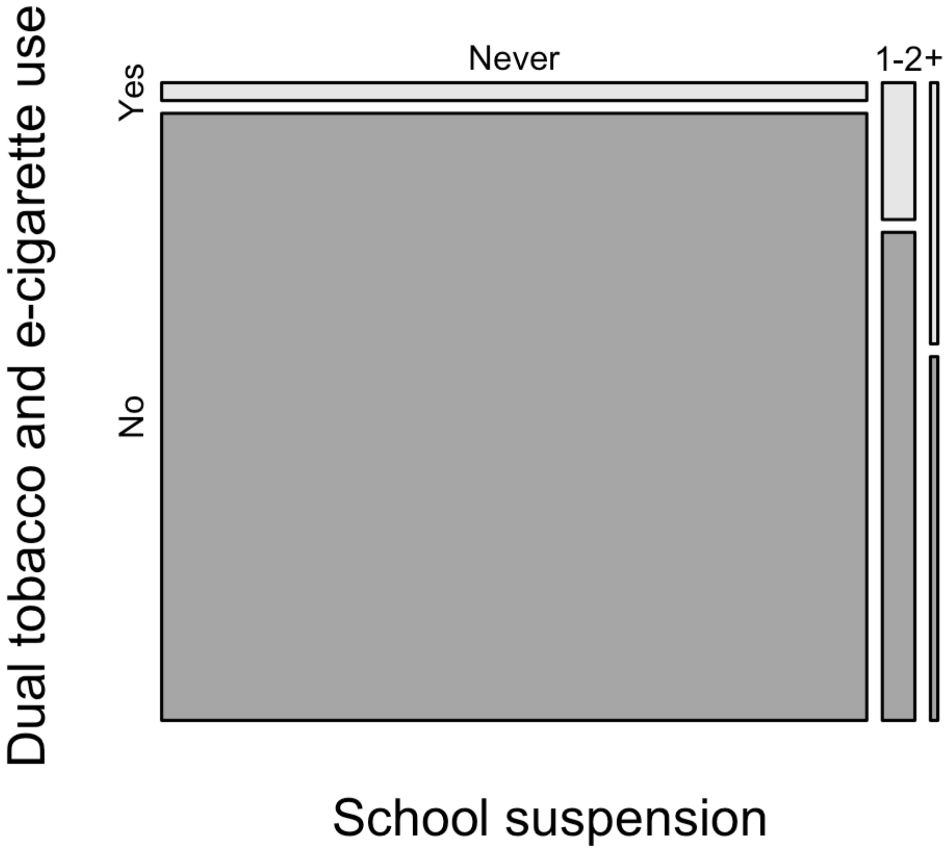

Fig S19. Cross-Tabulation Visualization Showing Proportion of Dual Lifetime Tobacco and E-Cigarette by Perceived Risk of Smoking Marijuana Regularly. G = Great Risk, M = Moderate Risk, S = Slight Risk, N = No Risk.

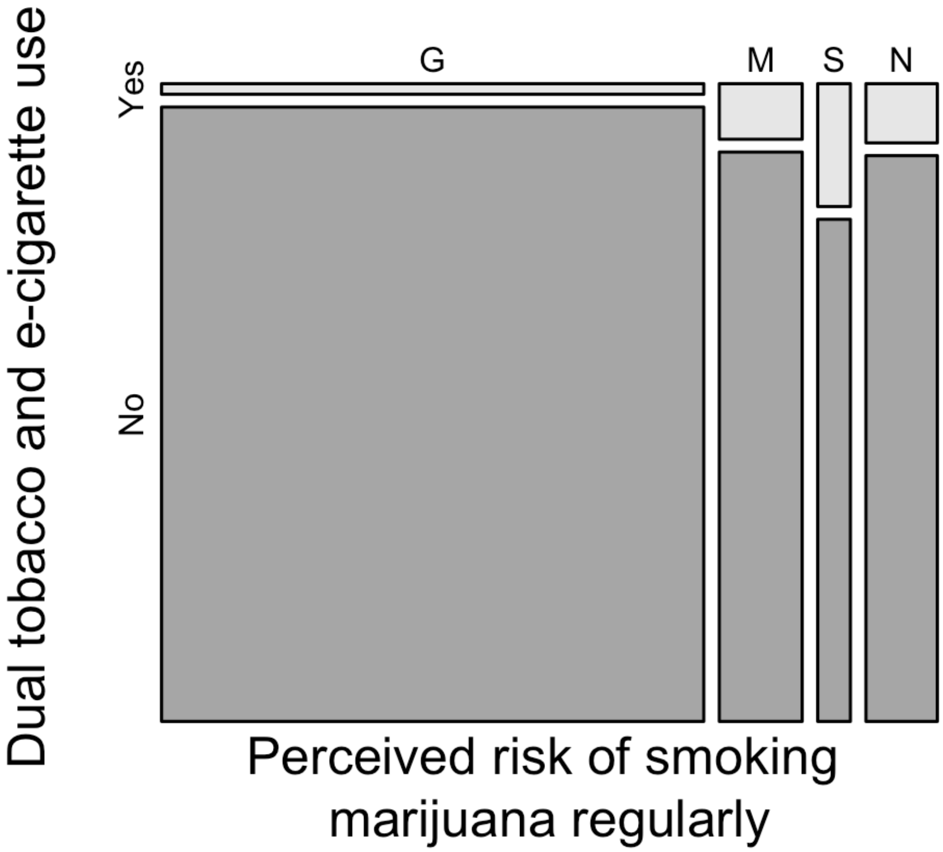

Supplement: S1 File — (PDF) [file pone.0287878.s001.pdf]
