# Supplementary material for: An ecological examination of early adolescent e-cigarette use: A machine learning approach to understanding a health epidemic
Source: PLoS One. 2024 Feb 14;19(2):e0287878. doi: 10.1371/journal.pone.0287878 (PMC10866513; doi:10.1371/journal.pone.0287878)
Supplement: S1 Table — (PDF) [file pone.0287878.s002.pdf]

SUPPLEMENTAL MATERIALS

Table S1. Variables included in machine learning analysis: Questions and responses.

**Short Description**	**Survey Questions**	**Responses**
**Demographics**		
Gender	Are you:	Male, Female
Age	How old are you?	Open-ended
Grade	What grade are you in?	6th, 7th, 8th, 9th, 10th, 11th, 12th
Race/Ethnicity	What is your race?	American Indian or Alaska Native, Asian, Black, Latinx, Pacific Islander, White
County	NA	NA
**Youth Rebelliousness**		
Does the opposite of what people tell them to do	I do the opposite of what people tell me, just to get them mad.	Very False, Somewhat False, Somewhat True, Very True
See how much I can get away with	I like to see how much I can get away with.	Very False, Somewhat False, Somewhat True, Very True
Ignore rules that get in their way	I ignore the rules that get in my way.	Very False, Somewhat False, Somewhat True, Very True
**Youth Antisocial behavior**	How many times in the past year (12 months) have you…	
Suspended from school	Been suspended from school?	Recode: Never, 1 or 2 Times, 3 or More Times
Carried a handgun	Carried a handgun?	Recode: Never, 1 or 2 Times, 3 or More Times
Sold illegal drugs	Sold illegal drugs?	Recode: Never, 1 or 2 Times, 3 or More Times
Stolen or tried to steal a motor vehicle	Stolen or tried to steal a motor vehicle such as a car or motorcycle?	Recode: Never, 1 or 2 Times, 3 or More Times
Arrested	Been arrested?	Recode: Never, 1 or 2 Times, 3 or More Times
Attacked someone to seriously hurt them	Attacked someone with the idea of seriously hurting them?	Recode: Never, 1 or 2 Times, 3 or More Times
Been drunk or high at school	Been drunk or high at school	Recode: Never, 1 or 2 Times, 3 or More Times
Taken a handgun to school	Taken a handgun to school	Recode: Never, 1 or 2 Times, 3 or More Times
**Youth Favorable attitudes towards antisocial behavior and drug use**	How wrong do you think it is for someone your age to…	
Attitude regarding taking a handgun to school	Take a handgun to school?	Very Wrong, Wrong, A Little Bit Wrong, Not Wrong at All
Attitude regarding stealing something	Steal anything worth more than $5?	Very Wrong, Wrong, A Little Bit Wrong, Not Wrong at All
Attitude regarding picking a fight with someone	Pick a fight with someone?	Very Wrong, Wrong, A Little Bit Wrong, Not Wrong at All
Attitude regarding attacking someone to seriously hurt them	Attack someone with the idea of seriously hurting them?	Very Wrong, Wrong, A Little Bit Wrong, Not Wrong at All
Attitude regarding skipping school without parents knowing	Stay away from school all day when their parents think they are at school?	Very Wrong, Wrong, A Little Bit Wrong, Not Wrong at All
Attitude regarding drinking alcohol regularly	Drink beer, wine or hard liquor (for example, vodka, whiskey or gin) regularly?	Very Wrong, Wrong, A Little Bit Wrong, Not Wrong at All
Attitude regarding smoking cigarettes	Smoke cigarettes?	Very Wrong, Wrong, A Little Bit Wrong, Not Wrong at All
Attitude regarding smoking marijuana	Smoke marijuana?	Very Wrong, Wrong, A Little Bit Wrong, Not Wrong at All
Attitude regarding using illegal drugs	Use LSD, cocaine, amphetamines or another illegal drug?	Very Wrong, Wrong, A Little Bit Wrong, Not Wrong at All
**Youth Substance use intentions**	Sometimes we don't know what we will do as adults, but we may have an idea. Please answer how true these statements may be for you. When I am an adult I will:	
Intentions to smoke cigarettes	Smoke cigarettes.	NO!, No, Yes, YES!
Intentions to drink alcohol	Drink beer, wine, or liquor.	NO!, No, Yes, YES!
Intentions to smoke marijuana	Smoke marijuana.	NO!, No, Yes, YES!
**Youth Perceived risk of drug use**	How much do you think people risk harming themselves (physically or in other ways) if they:	
Perceived risk of smoking one or more packs of cigarettes per day	Smoke one or more packs of cigarettes per day?	No Risk, Slight Risk, Moderate Risk, Great Risk
Perceived risk of trying marijuana once or twice a week	Try marijuana once or twice?	No Risk, Slight Risk, Moderate Risk, Great Risk
Perceived risk of smoking marijuana regularly	Smoke marijuana regularly?	No Risk, Slight Risk, Moderate Risk, Great Risk

Perceived risk of one or two drinks of alcohol nearly everyday	Take one or two drinks of an alcoholic beverage (beer, wine, liquor) nearly every day.	No Risk, Slight Risk, Moderate Risk, Great Risk
Perceived risk of abusing prescription drugs	How much do you think people risk harming themselves if they abuse prescription drugs?	No Risk, Slight Risk, Moderate Risk, Great Risk
Perceived risk of smoking one to five cigarettes per day	How much do you think people risk harming themselves (physically or in other ways) if they:	No Risk, Slight Risk, Moderate Risk, Great Risk
Perceived risk of e-cigarettes	How much do you think people risk harming themselves (physically or in other ways) if they use vape products such as e-cigarettes, vape pens, or mods?	No Risk, Slight Risk, Moderate Risk, Great Risk
**Youth Lifetime substance use**		
Lifetime tobacco use	Have you ever tried: cigarettes, even just one puff?	Recode: Yes, No
Lifetime alcohol use	On how many occasions (if any) have you had alcoholic beverages (beer, wine or hard liquor) to drink in your lifetime - more than just a few sips?	Recode: Yes, No
Lifetime marijuana use	How old were you when you first: used marijuana (grass, pot) or hashish (hash, hash oil).	Recode: Yes, No
Lifetime hallucinogen use	How old were you when you first: used LSD (acid) or other hallucinogens (like PCP, mescaline, peyote, “shrooms” or psilocybin.	Recode: Yes, No
Lifetime cocaine use	How old were you when you first: used cocaine (like cocaine powder) or “crack” (cocaine in chunk or rock form)?	Recode: Yes, No
Lifetime inhalants use	How old were you when you first: sniffed glue, breathed the contents of an aerosol spray can, or inhaled other gases or sprays, in order to get high?	Recode: Yes, No
Lifetime methamphetamine use	How old were you when you first: used methamphetamines (meth, speed, crank, crystal meth)	Recode: Yes, No
Lifetime prescription stimulants or amphetamines misuse	How old were you when you first: used prescription stimulants or amphetamines (such as Adderall, Ritalin, or Dexedrine) without a doctor telling you to take them?	Recode: Yes, No
Lifetime prescription sedatives misuse	How old were you when you first: used prescription sedatives including barbiturates or sleeping pills (such as phenobarbital, Tuinal, Seconal, Ambien, Lunesta, or Sonata) without a doctor telling you to take them?	Recode: Yes, No
Lifetime prescription tranquilizers misuse	How old were you when you first: used prescription tranquilizers (such as Librium, Valium, Xanax, Ativan, Soma, or Klonopin) without a doctor telling you to take them?	Recode: Yes, No
Lifetime narcotic prescription drug misuse	How old were you when you first: used narcotic prescription drugs (such as OxyContin, methadone, morphine, codeine, Demerol, Vicodin, Percocet) without a doctor telling you to take them?	Recode: Yes, No
Lifetime heroin use	How old were you when you first: used heroin?	Recode: Yes, No
Lifetime steroid use	On how many occasions (if any) have you used steroids or anabolic steroids (such as Anadrol, Oxandrin, Durabolin, Equipoise, or Depotesterone) in the past 30 days?	Recode: Yes, No
Lifetime synthetic marijuana use	On how many occasions have you used synthetic marijuana or herbal incense products (such as K2, Spice, or Gold) in the past 30 days?	Recode: Yes, No
**Community attachment**		
Would miss their neighborhood if they moved	If I had to move, I would miss the neighborhood I now live in.	NO!, No, Yes, YES!
Like their neighborhood	I like my neighborhood.	NO!, No, Yes, YES!
Would like to get out of their neighborhood	I’d like to get out of my neighborhood.	NO!, No, Yes, YES!
**Community rewards for prosocial involvement**		
Neighbors notice when they a good job and let them know	My neighbors notice when I am doing a good job and let me know about it.	NO!, No, Yes, YES!

Neighbors are proud of them when they do something well	There are people in my neighborhood who are proud of me when I do something well.	NO!, No, Yes, YES!
Neighbors encourage them to do their best	There are people in my neighborhood who encourage me to do my best.	NO!, No, Yes, YES!
**Community laws and norms**	How wrong would most adults in your neighborhood think it was for kids your age:	
Community attitudes regarding marijuana use	to use marijuana.	Very Wrong, Wrong, A little bit wrong, Not wrong at all
Community attitudes regarding alcohol use	to drink alcohol.	Very Wrong, Wrong, A little bit wrong, Not wrong at all
Community attitudes regarding smoking cigarettes	to smoke cigarettes.	Very Wrong, Wrong, A little bit wrong, Not wrong at all
Youth smoking marijuana in their neighborhood would be caught by police	If a kid smoked marijuana in your neighborhood would he or she be caught by the police?	NO!, No, Yes, YES!
Youth drinking alcohol in their neighborhood would be caught by police	If a kid drank some beer, wine, or hard liquor (for example, vodka, whiskey, or gin) in your neighborhood, would he or she be caught by the police?	NO!, No, Yes, YES!
Youth carrying a handgun in their neighborhood would be caught by police	If a kid carried a handgun in your neighborhood would he or she be caught by the police?	NO!, No, Yes, YES!
**Community perceived availability of substances**		
Perceived availability of cigarettes in their neighborhood	If you wanted to get some cigarettes, how easy would it be for you to get some?	Very Hard, Sort of Hard, Sort of Easy, Very Easy
Perceived availability of alcohol in their neighborhood	If you wanted to get some beer, wine, or hard liquor (for example, vodka, whiskey, or gin), how easy would it be for you to get some?	Very Hard, Sort of Hard, Sort of Easy, Very Easy
Perceived availability of other illegal drugs in their neighborhood	If you wanted to get a drug like cocaine, LSD, or amphetamines, how easy would it be for you to get some?	Very Hard, Sort of Hard, Sort of Easy, Very Easy
Perceived availability of marijuana in their neighborhood	If you wanted to get some marijuana, how easy would it be for you to get some?	Very Hard, Sort of Hard, Sort of Easy, Very Easy
Perceived availability of e-cigarettes in their neighborhood	If you wanted to get e-cigarettes, vape pens, or e-hookahs, how easy would it be for you to get some?	Very Hard, Sort of Hard, Sort of Easy, Very Easy
**Parent management**		
Parent ask about homework	My parents ask if I’ve gotten my homework done.	NO!, No, Yes, YES!
Parent know if they come home on time	Would your parents know if you did not come home on time?	NO!, No, Yes, YES!
Family rules are clear	The rules in my family are clear	NO!, No, Yes, YES!
Parent know where/who when out	When I am not at home, one of my parents knows where I am and who I am with.	NO!, No, Yes, YES!
Parent would catch alcohol drinking	If you drank some beer or wine or liquor (for example, vodka, whiskey, or gin) without your parents’ permission, would you be caught by your parents?	NO!, No, Yes, YES!
Family has clear rules about alcohol/drugs	My family has clear rules about alcohol and drug use.	NO!, No, Yes, YES!
Carried handgun without parent permission	If you carried a handgun without your parents’ permission, would you be caught by your parents?	NO!, No, Yes, YES!
Parents would catch skipping school	If you skipped school would you be caught by your parents?	NO!, No, Yes, YES!
**Family conflict**		
Family often insult or yell at each other	People in my family often insult or yell at each other.	NO!, No, Yes, YES!
Argue about the same things with family over and over	We argue about the same things in my family over and over.	NO!, No, Yes, YES!
Family have serious arguments	People in my family have serious arguments.	NO!, No, Yes, YES!
**Family history of substance abuse**		
Family member has had severe alcohol or drug problems	Has anyone in your family ever had severe alcohol or drug problems?	No, Yes
**Parental Attitudes Favorable Toward Drug Use and antisocial behavior**	How wrong do your parents feel it would be for YOU to:	Very Wrong, Wrong, A Little Bit Wrong, Not Wrong at All
Parent attitudes regarding their alcohol use	Drink beer, wine, or hard liquor (for example, vodka, whiskey, or gin) regularly?	Very Wrong, Wrong, A Little Bit Wrong, Not Wrong at All
Parent attitudes regarding their cigarette use	Smoke cigarettes?	Very Wrong, Wrong, A Little Bit Wrong, Not Wrong at All
Parent attitudes regarding their marijuana use	Smoke marijuana?	Very Wrong, Wrong, A Little Bit Wrong, Not Wrong at All

Parent attitudes regarding them stealing something	Steal anything worth more than $5?	Very Wrong, Wrong, A Little Bit Wrong, Not Wrong at All
Parent attitudes regarding them drawing graffiti	Draw graffiti, or write things, or draw pictures on buildings or other property (without the owner's permission)?	Very Wrong, Wrong, A Little Bit Wrong, Not Wrong at All
Parent attitudes regarding them picking a fight with someone	Pick a fight with someone?	Very Wrong, Wrong, A Little Bit Wrong, Not Wrong at All
Parent attitudes regarding their misuse of prescription drugs	How wrong do your parents feel it would be for YOU to use prescription drugs not prescribed to you?	Very Wrong, Wrong, A Little Bit Wrong, Not Wrong at All
Parent attitudes regarding their use of vape products	How wrong do your parents feel it would be for YOU to use vape products such as e-cigarettes, vape pens, or mods?	Very Wrong, Wrong, A Little Bit Wrong, Not Wrong at All
Parent attitudes regarding them drinking one or two alcoholic drinks a day	How wrong do your parents feel it would be for YOU to have one or two drinks of an alcoholic beverage nearly every day?	Very Wrong, Wrong, A Little Bit Wrong, Not Wrong at All
**Parental Reward for Prosocial Behavior**		
Enjoy spending time with their mother	Do you enjoy spending time with your mother?	NO!, No, Yes, YES!
Enjoy spending time with their father	Do you enjoy spending time with your father?	NO!, No, Yes, YES!
Parents notice when they are doing a good job and tell them	My parents notice when I am doing a good job, and let me know about it.	Never or Almost Never, Sometimes, Often, All the Time
Parents tell them they are proud of them for something they did	How often do your parents tell you they're proud of you for something you've done?	Never or Almost Never, Sometimes, Often, All the Time
**Number of antisocial and drug using best friends**	Think of you four best friends (the friends you feel closest to). In the past year (12 months), how many of your best friends have:	
Best friend suspended from school	Suspended From School	None, 1, 2, 3, 4
Best friend carried a gun	That Carried a Gun	None, 1, 2, 3, 4
Best friend sold drugs	That Sold Drugs	None, 1, 2, 3, 4
Best friend tried to steal cars	That Tried To Steal Cars	None, 1, 2, 3, 4
Best friend has been arrested	That Have Been Arrested	None, 1, 2, 3, 4
Best friend dropped out of school	That Dropped Our of School	None, 1, 2, 3, 4
Best friend some cigarettes	Smoked cigarettes?	None, 1, 2, 3, 4
Best friend tried alcohol	Tried beer, wine or hard liquor (for example, vodka, whiskey or gin) when their parents didn't know about it?	None, 1, 2, 3, 4
Best friend used marijuana	Used marijuana?	None, 1, 2, 3, 4
Best friend used LSD, cocaine, amphetamines or another illegal drugs	Used LSD, cocaine, amphetamines or another illegal drugs?	None, 1, 2, 3, 4
**Reward for antisocial behavior**	What are the chances you would be seen as cool if you:	
Social reward for smoking cigarettes	Smoked cigarettes?	No or Very Little Chance, Little Chance, Some Chance, Pretty Good Chance, Very Good Chance
Social reward for drinking alcohol regularly	Began drinking alcoholic beverages regularly, that is, at least once or twice a month?	No or Very Little Chance, Little Chance, Some Chance, Pretty Good Chance, Very Good Chance
Social reward for smoking marijuana	Smoked marijuana?	No or Very Little Chance, Little Chance, Some Chance, Pretty Good Chance, Very Good Chance
Social reward for carrying a handgun	Carried a handgun	No or Very Little Chance, Little Chance, Some Chance, Pretty Good Chance, Very Good Chance
**Rewards for prosocial involvement**	What are the chances you would be seen as cool if you:	
Social reward for working hard in school	Worked hard in school?	Very Good Chance, Pretty Good Chance, Some Chance, Little Chance, No or Very Little Chance
Social reward for defending someone verbally abused at school	Defended someone who was being verbally abused at school?	Very Good Chance, Pretty Good Chance, Some Chance, Little Chance, No or Very Little Chance

Social reward for volunteering to do community service	Regularly volunteered to do community service?	Very Good Chance, Pretty Good Chance, Some Chance, Little Chance, No or Very Little Chance
**Interaction with prosocial peers**	Think of your four best friends (the friends you feel closest to). In the past year (12 months), how many of your best friends have:	
Best friend participated in clubs, organizations and activities at school	Participated in clubs, organizations and activities at school?	None, 1, 2, 3, 4
Best friend made a commitment to stay drug-free	Made a commitment to stay drug-free?	None, 1, 2, 3, 4
Best friend try to do well in school	Tried to do well in school?	None, 1, 2, 3, 4
Best friend likes school	Liked school?	None, 1, 2, 3, 4
Best friend regularly attend religious services	Regularly attended religious services?	None, 1, 2, 3, 4
**Academic performance**		
Their grades are better than most kids in their class	Are your school grades better than the grades of most students in your class?	NO!, No, Yes, YES!
**School commitment**	Now, thinking back over the past year in school, how often did you…	
Enjoy being in school	Enjoy being in school?	Never, Seldom, Sometimes, Often, Almost Always
Hate being in school	Hate being in school?	Never, Seldom, Sometimes, Often, Almost Always
Try to do their best in school	Try to do your best work in school?	Never, Seldom, Sometimes, Often, Almost Always
Feel that school work is meaningful and important	How often do you feel that the school work you are assigned is meaningful and important?	Almost Always, Often, Sometimes, Seldom, Never
Things learned in school will be important later in life	How important do you think the things you are learning in school are going to be for your later life?	Very Important, Quite Important, Fairly Important, Slightly Important, Not at All Important
How interesting most of their courses are to them	How interesting are most of your courses to you?	Very Interesting & Stimulating, Quite Interesting, Fairly Interesting, Slightly Interesting, Not at All Interesting
Skipped school in the last four days	During the LAST FOUR WEEKS how many whole days of school have you missed because you skipped or "cut"	None, 1, 2, 3, 4-5, 6-10, 11 or More Days
**School environment**		
Teachers notice when they are doing a good job	My teachers notice when I am doing a good job and lets me know about it.	NO!, No, Yes, YES!
Feel safe in school	I feel safe at my school.	NO!, No, Yes, YES!
School tells parents when they do something well	The school lets my parents know when I have done something well.	NO!, No, Yes, YES!
Teachers praise them when they work hard in school	My teachers praise me when I work hard in school.	NO!, No, Yes, YES!
**Outcome**		
Lifetime cigarette use	Have you ever tried: electronic cigarettes or e-cigarette?	Yes, No
